# Incidence, Potential Mechanisms, and Clinical Significance of Cavernous Sinus Air Sign

**DOI:** 10.3390/diagnostics15030344

**Published:** 2025-01-31

**Authors:** Bo Kyu Kim, Sung-Hye You, Byungjun Kim

**Affiliations:** Department of Radiology, Anam Hospital, Korea University College of Medicine, #126-1, 5-Ka Anam-dong, Sungbuk ku, Seoul 136-705, Republic of Korea; stingray0379@naver.com (B.K.K.); cardillo@korea.ac.kr (B.K.)

**Keywords:** cavernous sinus, air embolism, computed tomography

## Abstract

**Background/Objectives:** The cavernous sinus air sign, historically linked to trauma or venous sinus thrombosis, has recently been reported in association with retrograde venous air embolism, often without clinical significance. Despite this, its exact prevalence, etiology, and clinical relevance remain unclear. This study aims to systematically evaluate the incidence of the cavernous sinus air sign in patients undergoing CT angiography (CTA) and to assess its potential clinical implications. **Methods:** We retrospectively analyzed data from patients who underwent CTA between January 2021 and December 2021. The cavernous sinus air sign was defined radiologically as air-density foci within the cavernous sinus, with Hounsfield units lower than those of orbital fat. Key variables included clinical indications for CTA, evidence of venous reflux of contrast media, the laterality of contrast injection, and the presence of brachiocephalic vein stenosis. Comparative analyses were performed to identify factors associated with the occurrence of the cavernous sinus air sign. **Results:** Among the 2,821 patients evaluated, the cavernous sinus air sign was identified in 35 cases (1.2%). Notably, none of these patients had a history of trauma or venous sinus thrombosis. Follow-up CT imaging was available for 27 of the 35 cases (77.1%), and in all instances, the cavernous sinus air sign resolved spontaneously. A statistically significant association was found between the cavernous sinus air sign and left-sided peripheral intravenous contrast injection, observed in 8.6% of affected patients compared to 1.5% in those without the sign (*p* = 0.001). Venous reflux into the internal jugular vein was also more frequent in patients with the air sign (34.3% vs. 14.1%, *p* = 0.001). These findings suggest a mechanical component, likely related to retrograde air embolism, influenced by anatomical and procedural factors. **Conclusions:** The isolated presence of the cavernous sinus air sign, in the absence of relevant clinical conditions, is most likely a benign, incidental finding associated with retrograde air embolism.

## 1. Introduction

The cavernous sinus air sign has been reported in various studies as a potential indicator of trauma, such as skull base fractures, and as being associated with serious conditions like barotrauma or septic thrombosis [1,2]. Skull base fracture is a well-documented risk factor for dural venous sinus thrombosis (DVST), and previous studies reported that the presence of venous sinus air is significantly associated with the development of DVST [3,4,5]. Delayed diagnosis and management of DVST following traumatic brain injury can result in catastrophic outcomes, further emphasizing the clinical importance of this radiological sign [6].

On the other hand, other studies have reported instances where air bubbles enter the venous sinus through mechanisms unrelated to trauma or pathology. For example, air bubbles have been observed migrating into the venous sinus via scalp veins during intravenous (IV) contrast injection, particularly when improper technique or elevated venous pressure is involved [7,8]. Additionally, cases of air embolism reaching the cavernous sinus through an IV line in healthy individuals in an upright position have been documented, further complicating the interpretation of this finding [9]. 

In this context, distinguishing between a pathological cause requiring urgent intervention and a benign or iatrogenic origin can be challenging. This finding is uncommon in most healthy individuals, and its true incidence remains uncertain due to the limited availability of large-scale studies. Moreover, variations in imaging modalities, patient positioning, and physiological conditions may affect the detection of venous sinus air, contributing to discrepancies in reported cases.

Previous studies have identified air bubbles in the dural venous sinus using non-contrast brain computed tomography (CT) scans. This modality is often the primary imaging tool in clinical practice due to its widespread availability, rapid acquisition time, and utility in acute settings [5,10,11]. Non-enhanced CT is highly sensitive for detecting air; however, it has relatively restricted spatial resolution with large slice intervals, and a limited ability to differentiate between air in the venous sinus and air in adjacent compartments, such as the subarachnoid space [11].

To address these limitations, contrast-enhanced techniques such as CT angiography (CTA) offer several advantages. CTA not only provides higher spatial resolution but also improves the differentiation between venous structures and the adjacent subarachnoid space. Furthermore, CTA enables the simultaneous evaluation of vascular anatomy, allowing clinicians to assess associated conditions like venous sinus thrombosis, venous stenosis, or anatomical variations. This study aims to assess the frequency and clinical significance of the cavernous sinus air sign using CTA, which integrates clinical and radiological data.

## 2. Materials and Methods

### 2.1. Patient Selection

This retrospective study was approved by the institutional review board of Korea University Anam Hospital, and the requirement for informed consent was waived (IRB No. 2024AN0551). We reviewed consecutive patients who underwent CTA between January 2021 and December 2021.

### 2.2. Imaging Protocol

All CT scans were performed using a 128-channel multidetector CT scanner (Somatom Definition AS+; Siemens, Erlangen, Germany). The scanning parameters were standardized at 100 kilovolts (kV) and 200 milliampere-seconds (mAs). For contrast-enhanced imaging, 90 mL of a non-ionic iodinated contrast agent (iobitridol, 350 mg iodine/mL) was administered intravenously at a rate of 5.0 mL/s. This was immediately followed by a 30 mL saline flush at the same rate to enhance uniform contrast distribution. Bolus triggering was employed with a region of interest set at the ascending aorta, and scanning commenced once the enhancement reached a threshold of 100 Hounsfield units.

The IV line was placed in accessible forearm veins, typically in the antecubital region, using a 20-gauge needle. In cases where antecubital veins were unavailable, other accessible forearm veins were utilized. Securing the IV line was performed preferentially on the right arm [12]. 

### 2.3. Image Interpretation

Two experienced neuroradiologists, with 13 and 11 years of clinical experience, independently evaluated all CT images. The evaluation was performed using thin-section axial images reconstructed at a thickness of 1.0 mm. The primary focus was to identify the presence of air in the cavernous sinus, defined as discrete foci of air density located within the contrast-filled cavernous sinus. These foci were characterized by Hounsfield units (HU) lower than those of orbital fat, ensuring reliable differentiation from other structures and artifacts.

In addition to detecting the cavernous sinus air sign, the neuroradiologists assessed for signs of thrombosis in the craniocervical venous system. The presence of air in venous structures outside the cavernous sinus, such as the internal jugular vein, inferior petrosal sinus, or other cranial venous pathways, was systematically documented.

To investigate the potential causes of venous air, particularly the reflux of air or contrast media from the subclavian vein to the ipsilateral internal jugular vein, inferior petrosal sinus, or cavernous sinus, specific details were examined. The location of the intravenous (IV) line at the time of imaging was recorded, along with the presence of contrast media within venous structures cranial to the subclavian vein. To combine detailed imaging analysis with clinical data, the presence of skull base fractures, history of blunt head trauma, and medical conditions indicating sepsis or other inflammatory conditions in the cervical area were also evaluated through medical records.

### 2.4. Statistical Analysis

All data in this study are reported either as numbers with their corresponding percentages or as means accompanied by standard deviations. Statistical analyses were conducted using appropriate methods based on the data type and distribution. Categorical variables were analyzed using the Chi-square test or Fisher’s exact test, depending on the suitability for the dataset. Continuous variables that followed a normal distribution were evaluated using Student’s *t*-tests. For all analyses, two-sided *p*-values were calculated, with values less than 0.05 considered statistically significant. Statistical analyses were performed using SPSS Statistics, version 22.0 (IBM Corporation, Armonk, NY, USA).

## 3. Results

A total of 2,821 patients were included in this study, all of whom underwent CTA for various clinical indications, as shown in Table 1. No patients had air density detection hindered by artifacts. Of these, 34 patients (1.2%) underwent CTA specifically to assess skull fractures or trauma involving the craniocervical region. Additionally, five patients (0.2%) presented with severe inflammatory conditions that affected the cavernous sinus region, either directly or indirectly. These cases included two patients with invasive fungal sinusitis extending to the cavernous sinus, two with cerebral septic emboli secondary to infective endocarditis, and one with temporal scalp thrombophlebitis.

**Figure 1:**
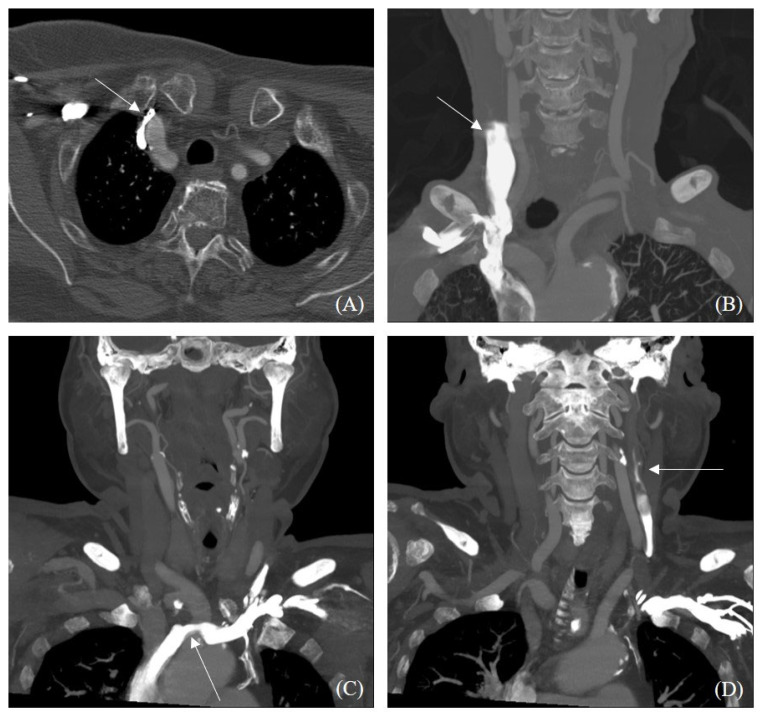
Venous reflux into internal jugular vein visible on CT angiography. Right brachiocephalic vein stenosis is observed due to tortuous course of brachiocephalic artery (**A**). Contrast reflux into ipsilateral internal jugular vein while administrating contrast media at peripheral vein at right forearm (**B**). Patient with left brachiocephalic vein stenosis with internal jugular vein reflux (**C**,**D**). The left brachiocephalic vein shows a longer and straighter course than the right brachiocephalic vein, and is more likely to be accompanied by stenosis between the aortic arch and sternum.

The cavernous sinus air sign was observed in 35 patients (1.2%). The clinical and imaging characteristics of these patients are summarized in Table 2. None of the patients with a positive cavernous sinus air sign had a history of trauma or severe inflammatory conditions. Among patients with the cavernous sinus air sign, a higher proportion had IV lines placed in the left arm (8.6% vs. 1.5%, *p* = 0.001). Reflux of contrast media into the ipsilateral internal jugular vein from the subclavian vein was more frequently observed in patients with the cavernous sinus air sign (34.3% vs. 13.9%, *p* = 0.001) (Figure 1). Of the 415 patients showing venous reflux, 57 (13.7%) had stenosis of the brachiocephalic vein on the contrast-injected side. Although brachiocephalic vein stenosis was more common in patients with the cavernous sinus air sign, the difference was not statistically significant (*p* = 0.099).

**Figure 2:**
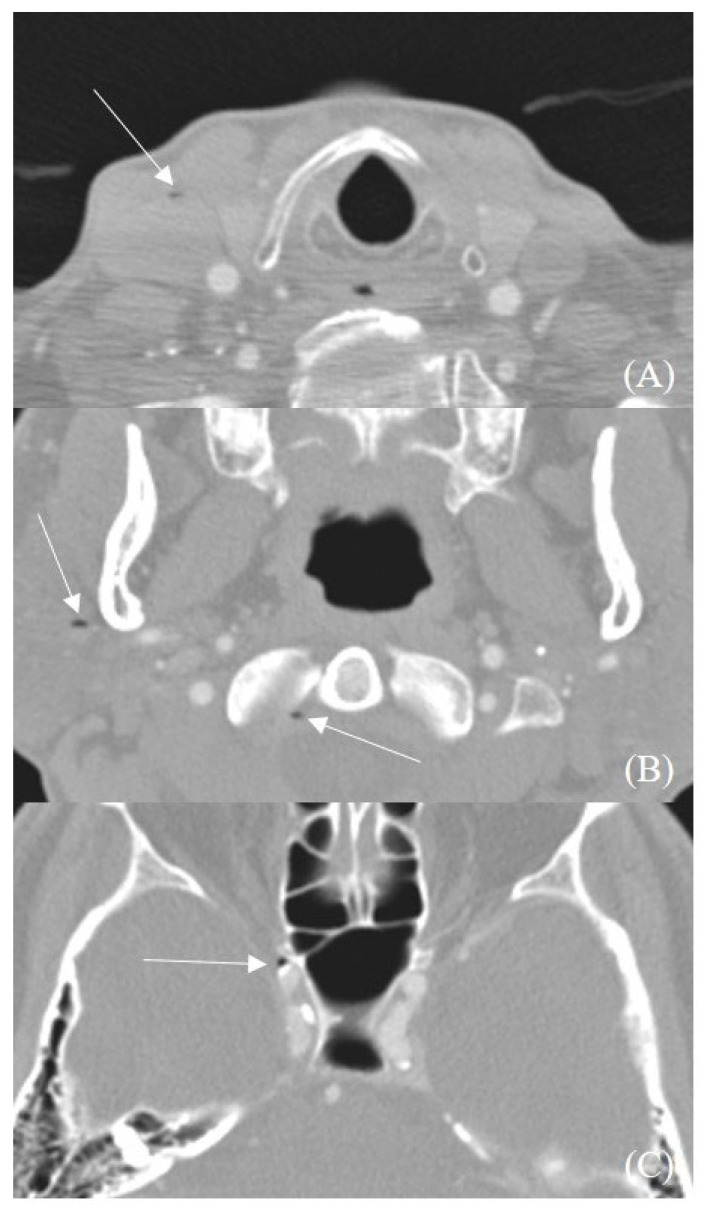
Retrograde air embolism into cavernous sinus. Multiple air bubbles (white arrows) can be observed at internal jugular vein (**A**), external jugular vein and inferior petrosal sinus (**B**), and cavernous sinus (**C**). It shows that air bubbles reflux along the path where the cavernous sinus normally drains venous blood into the heart.

Among the patients with the cavernous sinus air sign, three also exhibited air densities in other venous structures, highlighting the potential systemic distribution of venous air emboli. Specifically, two patients exhibited air densities in the internal jugular vein, while one patient showed air densities in both the internal and external jugular veins, as illustrated in Figure 2. 

**Figure 3:**
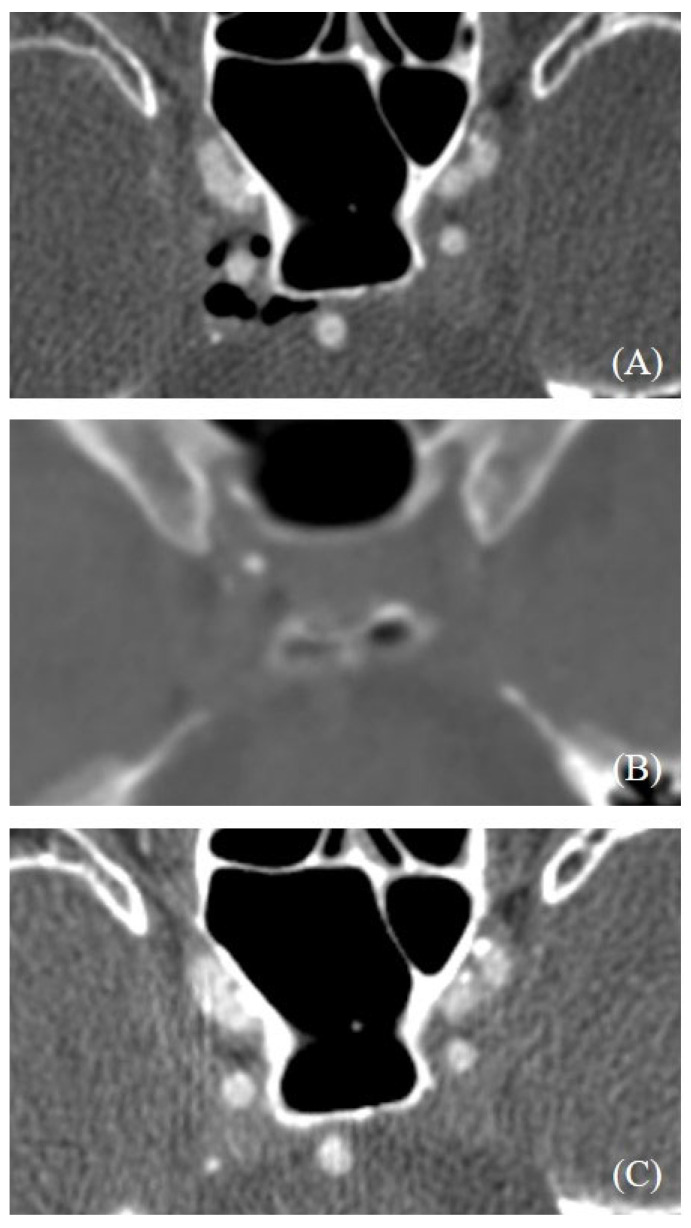
Initial CT angiography showed the cavernous sinus air sign in right cavernous sinus (**A**). Follow-up brain CT after 1 day (**B**) and CT angiography performed 7 days after the initial CTA (**C**) showed spontaneous disappearance of cavernous sinus air sign.

Follow-up brain CT imaging was available for 27 of the 35 patients (77.1%) who initially showed the cavernous sinus air sign. Remarkably, in all cases, the air densities within the cavernous sinus resolved spontaneously, without requiring any specific medical or surgical intervention (Figure 3).

## 4. Discussion

In this study, the cavernous sinus air sign was identified as an infrequent phenomenon, observed in approximately 1.2% of the evaluated cases. Its occurrence was notably linked to a range of nonspecific clinical conditions, rather than to well-known pathological states such as trauma or cavernous sinus thrombosis. Interestingly, even among patients who had experienced traumatic events, no significant correlation was found between trauma and the manifestation of the cavernous sinus air sign. This finding suggests that its appearance is not a reliable indicator of traumatic injury or severe inflammatory pathology. Instead, it may reflect incidental or less critical physiological or pathological changes, emphasizing the need for careful clinical evaluation to avoid overinterpretation. These observations pave the way for further research into its etiology, potential clinical implications, and diagnostic relevance, with the aim of clarifying whether it represents a benign anomaly or holds subtle diagnostic value in specific contexts.

Our study specifically examined patients undergoing CT angiography, enabling precise differentiation between pneumocephalus—characterized by air in the subarachnoid space—and air confined to the cavernous sinus. Notably, all patients in the study had a peripheral venous line in place for contrast injection, a factor that seems to play a pivotal role in the observed phenomenon. Based on our findings, the majority of cases involving the cavernous sinus air sign are likely caused by venous air emboli introduced during the IV injection process. Furthermore, air densities identified on CT in the context of skull base fractures or penetrating trauma may indicate extravascular sources of air. These results highlight the importance of contextual evaluation in distinguishing between benign procedural artifacts and clinically significant findings, particularly in scenarios involving trauma or other complicating factors [10].

Cannulation of central or scalp veins has long been considered a potential mechanism for introducing air bubbles into the cavernous sinus, as suggested by prior studies [13,14]. The absence of venous valves in scalp veins increases the likelihood of air entry into the cavernous sinus. This anatomical vulnerability highlights the importance of careful technique in such settings [15,16]. By contrast, air embolism is traditionally considered less common with peripheral IV lines. However, even when a venous valve is present in the internal jugular vein, reverse flow is often observed on Doppler ultrasound, demonstrating the complexity of venous flow dynamics [17]. 

This phenomenon is particularly notable in the left internal jugular vein, where reflux is often linked to anatomical factors, such as narrowing of the left brachiocephalic vein [18,19]. The left brachiocephalic vein follows a longer and more horizontal course than the right, traversing between the sternum and the aortic arch or its major branches. This path places the vein at risk of compression within the retrosternal space, which can lead to focal stenosis. Such compression is commonly associated with venous reflux, increasing the risk of complications such as air embolism when IV lines are placed on the left. In this context, left-sided IV line placement for contrast medium injection is generally avoided, especially in cases requiring precise venous flow control [20]. In our cohort, however, 45 patients (1.6%) had left-sided IV line access due to inaccessible right-sided veins. These cases demonstrated an increased susceptibility to venous reflux and air embolism. Moreover, internal jugular vein reflux is often observed in hypertensive patients, a phenomenon linked to elevated central venous pressure that can exacerbate retrograde flow [21]. However, venous reflux is not exclusive to hypertensive individuals; it can also occur in normotensive patients due to the variability of factors influencing venous flow, such as transient changes in thoracic pressure and anatomical variations. 

Various anatomical factors, such as mediastinal masses, aortic aneurysms, vascular anomalies, superior vena cava syndrome, or severe congestive heart failure, can also contribute to venous reflux. Elderly patients with an ectatic aortic arch also have a higher chance of brachiocephalic vein compression [22]. In our study, only two patients with the cavernous sinus air sign exhibited focal narrowing of the brachiocephalic vein, which was clinically insignificant. This finding suggests that venous reflux can occur without significant anatomical factors and may instead result from high-pressure injection of contrast media [23].

A previous study using time-of-flight MR angiography reported increased signal intensity in the inferior petrosal and cavernous sinuses due to retrograde flow, a phenomenon observed not only in cases of brachiocephalic vein stenosis but also during the Valsalva maneuver or breath-holding [18,24]. Thus, venous reflux can be influenced by both anatomical and physiological factors.

Furthermore, Uchino et al. [25] reported that venous reflux is more frequently observed in the inferior petrosal sinus than in the transverse or sigmoid sinuses on time-of-flight MR angiography. Nevertheless, venous reflux involving the transverse or sigmoid sinuses has also been documented in multiple studies [22,26]. Our study found that air embolism was more commonly identified in the cavernous sinus. One plausible explanation for this disparity is related to the positional dynamics during imaging. In the supine position, air bubbles in non-dependent areas of the venous system are more likely to migrate into the internal jugular vein and subsequently enter the cavernous sinus via the inferior petrosal sinus. However, the venous drainage system is highly variable, with considerable anatomical differences between individuals. These variations complicate efforts to predict the precise pathways of venous reflux using noninvasive techniques [27,28]. The air densities observed within venous structures, illustrated in Figure 2, provide potential clues supporting this hypothesis.

Our study demonstrated that clinically insignificant, small air bubbles spontaneously disappear on follow-up imaging. Conservative management of pneumocephalus includes head elevation, bed rest, avoidance of coughing, nose blowing, or the Valsalva maneuver [29]. In all of our cases with the cavernous sinus air sign, the amount of air was small and may eventually be absorbed in the capillary bed. It seems that, without other explanatory signs such as trauma or infection, small air bubbles in the cavernous sinus do not require treatment and appear to be absorbed naturally. However, another possible mechanism for cavernous sinus air sign is that air may enter the cavernous sinus from the sphenoid sinus due to a skull base fracture, or may result from gas-forming organisms. Therefore, the cavernous sinus air sign should be interpreted in conjunction with the accompanying clinical and radiologic context, rather than being considered pathological on its own.

This study has several notable limitations that warrant discussion. First, the retrospective design inherently limits the scope of the analyses. Second, all patients included in this study underwent intravenous line manipulation for contrast media injection. Also, patients with suspected trauma or a septic condition in the emergency room had an IV line secured prior to CT acquisition, so evaluation of the cavernous sinus air sign in the absence of an IV line was not possible. When the cavernous sinus air sign is detected without an IV line, underlying trauma or sinus thrombosis should be carefully considered as potential contributors. Additionally, the incidence of venous reflux and the cavernous sinus air sign, may be exaggerated by the forceful injection of contrast media. It is also worth noting that venous air embolism can occur even without contrast injection, solely due to the presence of an IV line [10]. Third, the protocol for brain CTA in this study did not include breath-holding, which could influence the likelihood of venous reflux or the cavernous sinus air sign. Previous research has demonstrated that inspiration can alter the distance between the aortic arch and the sternum, as well as the diameter of the brachiocephalic vein, potentially affecting venous flow dynamics [30]. Furthermore, this study did not evaluate factors such as internal jugular vein valve insufficiency, cardiac output, or blood pressure during CTA, all of which may contribute to the occurrence of the cavernous sinus air sign. While underlying hypertension was found to have no significant effect in this study, interpreting this result is challenging given the variability in blood pressure measurements. Recent studies utilizing advanced imaging techniques, such as pseudo-continuous arterial spin labeling, have suggested a relationship between retrograde flow in the internal jugular vein and hypertension, highlighting the need for further exploration in this area [21]. Finally, while the amount of air embolism observed in our study was small, the clinical significance of larger volumes of venous air embolism remains an area not specifically investigated. Previous studies have suggested that venous air embolism, particularly in significant amounts, can be a potentially life-threatening condition with the capacity to cause severe complications such as cardiovascular collapse, pulmonary embolism, and neurological impairment [31]. While a small amount of air may not pose an immediate threat, care must be taken to expel air during IV line manipulation.

In conclusion, the isolated presence of the cavernous sinus air sign appears to be a benign and incidental finding, likely resulting from intravenous air reflux. This sign should be interpreted with attention to the clinical context.

## Figures and Tables

**Table 1:** Clinical indications for CT angiography.

Indication	Number
Stroke evaluation	686 (24.3%)
Trauma evaluation	57 (2.0%)
Unruptured intracranial aneurysm	340 (12.1%)
Postoperative follow up	171 (6.1%)
Neurologic symptom (dizziness, headache, syncope, seizure, etc.)	738 (26.1%)
Atherosclerotic vascular disease	490 (17.4%)
Cerebral venous thrombosis	4 (0.1%)
Moyamoya disease	9 (0.3%)
SAH	68 (2.4%)
Trauma	34 (1.2%)
Nontraumatic ICH, IVH, SDH	140 (5.0%)
Severe inflammatory condition	5 (0.2%)
Preoperative evaluation	79 (2.8%)

ICH: intracerebral hemorrhage; IVH: intraventricular hemorrhage; SAH: subarachnoid hemorrhage; SDH: subdural hemorrhage.

**Table 2:** Clinical and imaging characteristics for patients with cavernous sinus air sign.

	Number		*p*-Value
Cavernous Sinus Air Sign	Positive (*n* = 35)	Negative (*n* = 2786)	
Clinical indication			
Stroke evaluation	11 (31.4%)	686 (24.6%)	0.861
Unruptured intracranial aneurysm	8 (22.9%)	340 (12.2%)	0.057
Neurologic symptom	12 (34.3%)	738 (26.5%)	0.300
Postoperative follow-up	4 (11.4%)	171 (6.1%)	0.197
Age	64.8 ± 18.4	64.0 ± 14.8	0.783
Female	13 (37.1%)	1503 (53.9%)	0.060
Hypertension	15 (42.9%)	1098 (39.4%)	0.679
Peripheral intravenous line			0.001
Right	32 (91.4%)	2744 (98.5%)	
Left	3 (8.6%)	42 (1.5%)	
Air in other than the cavernous sinus			0.001
Absent	32 (91.4%)	2776 (99.7%)	
Present	3 (8.6%)	10 (0.3%)	
Venous reflux			0.001
Absent	23 (65.7%)	2393 (85.9%)	
Present	12 (34.3%)	393 (14.1%)	
Brachiocephalic vein stenosis	2 (5.7%)	52 (1.9%)	0.099

## Data Availability

All data relevant to the study are included in the article.
